# How the unvaccinated threaten the vaccinated for COVID-19: A Darwinian perspective

**DOI:** 10.1073/pnas.2114279118

**Published:** 2021-09-20

**Authors:** Emanuel Goldman

**Affiliations:** ^a^Department of Microbiology, Biochemistry and Molecular Genetics, New Jersey Medical School, Rutgers, The State University of New Jersey, Newark, NJ 07103

Imai et al. (1) have characterized yet another variant of severe acute respiratory syndrome coronavirus 2 (SARS-CoV-2), the virus responsible for COVID-19, this one originating in Brazil. The good news is that it appears that vaccines currently available are still expected to provide protection against this variant. However, what about the next variant, one we have not seen yet? Will we still be protected?

In 1859, Charles Darwin published *On the Origin of Species* (2), in which he outlined the principles of natural selection and survival of the fittest. The world presently has the unwelcome opportunity to see the principles of evolution as enumerated by Darwin play out in real time, in the interactions of the human population with SARS-CoV-2. The world could have easily skipped this unpleasant lesson, had there not been such large numbers of the human population unwilling to be vaccinated against this disease.

SARS-CoV-2 has shown that it can mutate into many variants of the original agent (3). An unvaccinated pool of individuals provides a reservoir for the virus to continue to grow and multiply, and therefore more opportunities for such variants to emerge. When this occurs within a background of a largely vaccinated population, natural selection will favor a variant that is resistant to the vaccine.

So far, we have been lucky that the variants that have emerged can still be somewhat controlled by current vaccines, probably because these variants evolved in mostly unvaccinated populations and were not subject to selective pressure of having to grow in vaccinated hosts. Nevertheless, the Delta variant is exhibiting increased frequency of breakthrough infections among the vaccinated (4).

The real danger is a future variant, which will be the legacy of those people who are not getting vaccinated providing a breeding ground for the virus to continue to generate variants. A variant could arise that is resistant to current vaccines, rendering those already vaccinated susceptible again.

Progress we have made in overcoming the pandemic will be lost. New vaccines will have to be developed. Lockdowns and masks will once again be required. Many more who are currently protected, especially among the vulnerable, will die.

This dire prediction need not occur if universal vaccination is adopted, or mandated, to protect everyone, including those who are already vaccinated.

Darwinian selection may also yet solve the problem with a much crueler calculus. The unvaccinated will either get sick and survive, and therefore be the equivalent of vaccinated, or they will die and therefore be removed as breeding grounds for the virus.

The National Archives in the United Kingdom note that, in 1665, during the Black Death plague, “to prevent the disease spreading, a victim was locked in their house with their entire family, condemning them all to death” (5). Vaccinations offer a much more humane response to prevent spread of this disease. The path forward is in the hands of the unvaccinated, and in the political will of the authorities.
